# Polyvalent Phage CoNShP-3 as a Natural Antimicrobial Agent Showing Lytic and Antibiofilm Activities against Antibiotic-Resistant Coagulase-Negative Staphylococci Strains

**DOI:** 10.3390/foods9050673

**Published:** 2020-05-23

**Authors:** Ahmed R. Sofy, Naglaa F. Abd El Haliem, Ehab E. Refaey, Ahmed A. Hmed

**Affiliations:** 1 Botany and Microbiology Department, Faculty of Science, Al-Azhar University, Nasr City, 11884 Cairo, Egypt; ehabrefaey@azhar.edu.eg; 2 Microbiology and Immunology Department, Faculty of Medicine (Girls), Al-Azhar University, Nasr City, 11884 Cairo, Egypt; nagllafarouk.micro@azhar.edu.eg

**Keywords:** natural antimicrobials, food safety, bacteriophages, CoNShP-3, phage biocontrol, foodborne bacteria, coagulase-negative *Staphylococcus*, CoNS, antibiotic-resistance, antibiofilm activity

## Abstract

Synthetic antimicrobials have a negative impact on food quality and consumer health, which is why natural antimicrobials are urgently needed. Coagulase-negative staphylococci (CoNS) has gained considerable importance for food poisoning and infection in humans and animals, particularly in biofilms. As a result, this study was conducted to control the CoNS isolated from food samples in Egypt. CoNS isolates were selected on the basis of their antibiotic susceptibility profiles and their biofilm-associated behavior. In this context, a total of 29 different bacteriophages were isolated and, in particular, lytic phages (6 isolates) were selected. The host range and physiological parameters of the lytic phages have been studied. Electron microscopy images showed that lytic phages were members of the families *Myoviridae* (CoNShP-1, CoNShP-3, and CoNSeP-2 isolates) and *Siphoviridae* (CoNShP-2, CoNSsP-1, and CoNSeP-1 isolates). CoNShP-1, CoNShP-2, and CoNShP-3 were found to be virulent to *Staphylococcus haemolyticus*, CoNSsP-1 to *Staphylococcus saprophyticus* and CoNSeP-1 and CoNSeP-2 to *Staphylococcus epidermidis*. Interestingly, the CoNShP-3 exhibited a typical polyvalent behavior, where not only lysis CoNS, but also other genera include *Staphylococcus aureus*, methicillin-resistant *Staphylococcus aureus* (MRSA), vancomycin-resistant *Staphylococcus aureus* (VRSA), *Bacillus cereus* and *Bacillus subtilis*. In addition, CoNShP-3 phage showed high stability at different temperatures and pH levels. Indeed, CoNShP-3 phage showed an antibiofilm effect against *Staphylococcus epidermidis* CFS79 and *Staphylococcus haemolyticus* CFS43, respectively, while *Staphylococcus saprophyticus* CFS28 biofilm was completely removed. Finally, CoNShP-3 phage demonstrated a high preservative efficacy over short and long periods of storage against inoculated CoNS in chicken breast sections. In conclusion, this study highlights the control of CoNS pathogens using a polyvalent lytic phage as a natural antibacterial and antibiofilm agent from a food safety perspective.

## 1. Introduction

Bacterial contamination-related food poisoning is the leading cause of morbidity and mortality in developing countries [1]. Coagulase-positive staphylococci (CoPS) as *Staphylococcus aureus* produces staphylococcal enterotoxin (SE) and is responsible for almost all staphylococcal food poisoning [2,3]. The enterotoxigenic influence of coagulase-negative staphylococci (CoNS) in food intoxication has also been detected and increased [4]. CoNS are one of the leading causes of many infections and diseases, including nervous system infection, heart inflammation, and urinary tract infection (UTI) [5]. Several outbreaks of multidrug-resistant CoNS have been reported in intensive care units (ICUs) [6,7]. Moreover, it may be the cause of significant economic losses [8]. CoNS biofilm formation significantly affects their persistence, both on inanimate surfaces and in biological tissues, because the bacterial biofilm makes it to more adherent and shows resistance to disinfectants, biocides, and antibiotics [9,10]. For neonatal ICUs, in particular, it has been found that antibiotic-resistant *S. epidermidis* and *S. haemolyticus* strains that form biofilms lead to aggregation and, therefore, cause disease among neonates [11]. Such unwanted bacteria can be found in many foods, including processed meat products [12] and dairy products [13]. Preventing these foodborne bacteria and other food spoilage is usually done through traditional synthetic antimicrobials [14]. The need for natural biocontrol agents is expected to increase positively in the exchange of traditional synthetic antimicrobials with varying efficacy and negative impacts on food quality and consumer health [15,16,17,18]. Moreover, traditional synthetic compounds act not only against foodborne pathogens, but also against consumers’ microbiome [19]. Researchers and scientists should therefore devote their efforts to finding alternative solutions to this concern.

Bacteriophages are considered “smart antimicrobials”, infect target pathogenic bacteria with no effect on the commensal flora due to their specific nature [20,21]. They are therefore seen as an interesting way to replace traditional antibacterial products; indeed, phage biocontrol has become necessary because it is natural and safe and is considered to be green technology that is environmentally friendly [20,21,22]. Phage biocontrol is an incredibly attractive way to further improve the health of our food through the biological properties of lytic bacteriophages [22,23]. In this regard, several researchers have demonstrated the efficacy of the use of bacteriophage in different foods in order to minimize contamination [22,24,25,26]. Some bacteriophages are capable of withstanding high osmotic pressure, heat up to 60 °C, freeze, pH of 5.0, and disrupt some bacterial biofilms [27,28,29,30,31,32,33,34]. Despite all the advantages mentioned above, one of the main disadvantages of phage as a biocontrol agent is that the different components of the food matrix, such as fats, proteins, and carbohydrates, have limited effects on the ability of the bacteriophage to interact with its particular pathogens [22,35]. Moreover, the acceptance of the market may pose a challenge to the wider use of bacteriophage for the biocontrol of foodborne pathogens in food [22,23]. As a final consideration, phages are specialized in infection for their hosts, so that if food products appear to be contaminated with two or more foodborne bacterial pathogens, a single pathogen-specific phage preparation would not be successful in eliminating non-target pathogenic bacteria from food [22]. On the other hand, other phages are polyvalent (i.e., not host-specific) and have the ability to infect many bacteria of different genera [36,37,38,39].

The overall objective of this research is therefore to isolate the polyvalent lytic phage used as an alternative natural antibacterial agent in order to meet the food safety strategy by controlling CoNS resistant strains isolated from food and disrupting their biofilms.

## 2. Materials and Methods 

### 2.1. Bacterial Strains

#### 2.1.1. Source of CoNS Isolates

A total of 137 presumptive CoNS previously isolated from samples of white cheese and meat products according to Pamuk, et al. [40] and Soares Casaes Nunes, et al. [41]. Typical CoNS colonies were purified and kept at −80 °C in trypticase soy broth (TSB, DifcoTM, MI, USA) medium provided with 45% *v*/*v* glycerol for further studies.

#### 2.1.2. Inoculums Preparation of Isolates

The lawn strains were cultured into TSB medium and incubated at 37 °C until a mid-logarithmic phase. The concentration of the host inoculum was adjusted using CFU/mL compared to 0.5 McFarland turbidity standard (1.5 × 10^8^ CFU/mL) [42].

#### 2.1.3. Primary Identification of CoNS Isolates

Presumptive CoNS isolates were morphologically examined (non-motile, smooth, non-spore forming cocci, arranged in pairs, tetrad, grape-like cluster or occur singly) on Mannitol Salt Agar (DifcoTM, BD, Franklin Lakes, NJ, USA) and blood agar base (7% sheep blood) media. After an incubation period of 24–48 h at 37 °C, the morphological characteristics of the CoNS colonies were examined. In addition, common biochemical tests (Gram-stain, coagulase “tube test” and catalase) were performed according to Holt, et al. [43], Collins and Lyne [44] and Cheesbrough [45]. Based on morphological and biochemical characteristics, 93 of the 137 isolates represent the population of the CoNS.

#### 2.1.4. In Vitro Antibiotic Susceptibilities of CoNS Isolates

The antibiotic-resistance profiles of CoNS were performed for isolates that were identified primarily, in order to select the multi-drug resistant isolates for further studies. The test was performed according to Kirby–Bauer disk diffusion method [46]. In this assay, 16 antibiotics discs (Oxoid, UK) of different groups were used as follows: Erythromycin (15 mcg), Kanamycin (30 mcg), Tobramycin (10 mcg), Amoxicillin/clavulanic acid AMC (20 μg), Aztreonam (1 mcg), Rifamycin (30 mcg), Gentamicin (10 mcg), Streptomycin (10 μg), Cephradine (30 mcg), Tetracycline (30 mcg), Ciprofloxacin (5 mcg), Oxacillin (1 mcg), Ampicillin (10 mcg), Flucloxacillin (5 mcg), Clindamycin (2 mcg), and Levofloxacin (5 mcg). The results were interpreted by the National Committee for Clinical Laboratory Standards (NCCLS) protocols, and designated as R (resistant), I (intermediate sensitive), and S (sensitive) [47].

#### 2.1.5. Secondary Identification of CoNS Isolates

Bacterial isolates that were initially identified as CoNS and classified as the highest resistance isolates (*n* = 27/93; 29%) are then automatically confirmed by Biomerieux VITEK 2 identification system according to Bannerman, et al. [48] and Funke and Funke-Kissling [49].

#### 2.1.6. CoNS Strains for Phages Isolation 

Twenty-seven CoNS multi-drug resistant (MDR) strains resulting from the previous test were used to isolate the bacteriophages and other physiological parameters. These strains included *Staphylococcus haemolyticus* (*n* = 9/27; 33.3%), *Staphylococcus saprophyticus* (*n* = 7/27; 25.9%), *Staphylococcus epidermidis* (*n* = 7/27; 25.9%) and *Staphylococcus hominis* (*n* = 4/27; 14.8%).

#### 2.1.7. Biofilm Characteristics of MDR-CoNS Strains

Biofilm production activity of CoNS strains was quantitatively determined using the tissue culture plate (TCP) method in accordance with Bekir, et al. [50]. The assay was performed in a 96-well flat-bottom polystyrene microtiter plate (Sigma-Aldrich, Costar, USA) as follows: for all MDR-CoNS strains, 0.2 mL of bacterial suspension (2 × 10^6^ CFU/mL) was added to TSB with 0.25% glucose for each well (except background wells). The plate was then incubated in a shaker at 37 °C overnight, after which the wells were aspirated and washed using 0.2 mL/well phosphate-buffered saline (PBS) (pH = 7.2). The 95% ethanol was added to all wells (200 μL/well) to fix the adsorbed bacterial cells in the polystyrene wells. The biofilm was stained with 0.1% crystal violet (125 μL/well) (Sigma, USA) for 20 min, followed by washing the plate with PBS, and the dye was solubilized in 1% *w*/*v* SDS. The optical density was determined using ELISA reader (Sunrise™-TECAN, Switzerland) at optical density 570 nm (OD_570 nm_). The assay was performed in triplicate. The formed biofilm of the CoNS strains tested was identified as strong, moderate, or low, according to the criteria of Stepanović, et al. [51].

### 2.2. Detection of CoNS Bacteriophages 

#### 2.2.1. Source of Phages Isolation 

Thirty different samples of sewage water (20 mL) were collected from the Wastewater Treatment Plant (WWTP), (Kafr El-Sheikh governorate, Egypt) to isolate bacteriophages.

#### 2.2.2. Phages Isolation and Enrichment

In the TSB medium, a 10 mL of sewage water sample was enriched in equal volumes. An overnight culture of 100 μL for each strain of MDR-CoNS was then inoculated into the previous mixture in a separated sterile flask for each strain. The flasks were placed in shaker incubator (270 rpm) at 37 °C for 16–20 h. The mixtures were centrifuged (7000× *g*) for 20 min at 4 °C, followed by filtration using sterile disposable filters (0.45 μm), then transferred to sterile clean flasks and stored 4 °C [52].

#### 2.2.3. Phage Determination

##### Spot-Test Technique

Spot testing was performed according to Capra, et al. [53] as follows: 4 mL of TSB containing 0.7% agar was inoculated with 100 μL overnight culture of each MDR-CoNS host strain for the preparation of the soft layer. In trypticase soy agar (TSA) plates, the soft layer was added directly and left to about 15 min for solidification (overlay layer). To detect phage activity, 10 μL of the previous enriched samples were spotted on the surface of the soft layer and incubated overnight at 37 °C. Partial or complete lysis zones on TSA plates were detected after the incubation period. Lysis zones were then transferred separately using a sterilized wire loop to CM phage buffer (0.735 gm/L CaCl_2_. 2H_2_O: 2.5 g/L MgSO_4_. 7H_2_O; 0.05 g/L gelatin; 6 mL/L 1 M Tris buffer; pH 7.2).

##### Plaque Assay Technique

Phages plaques formation were assayed by overlay technique, according to Kaur, et al. [54] and Sangha, et al. [55]. Ten-fold serial dilutions for each phage lysate were performed. Each dilution of phage lysate (100 μL) was mixed with overnight culture host in the separate tube and incubated at 37 °C for 24 h. After mixing the tubes, 4 mL of soft agar layer was added into the previous suspension and immediately poured onto TSA plates, then incubated at 37 °C for 24 h. Plaques of different sizes and shapes were picked up and transferred in 1 mL CM phage buffer and left for 24 h at 25 °C to permits phages diffusion into the buffer.

#### 2.2.4. Phages Purification, Propagation, and Titration

Purification and propagation of phages were performed by the overlay method, according to Kaur, Harjai and Chhibber [54] and Sangha, Kumar, Agrawal, Deka and Verma [55]. A single plaque was picked up from the previous plates; the overlay procedure was repeated three times in succession in order to purify isolated phages. For phages propagation; 100 μL of the original CoNS lawn was mixed with 100 μL of phage suspension in TSB medium and incubated at 37 °C for 24 h. Ten overlay agar plates were prepared for each isolated phage, and 3 mL of CM phage buffer was added to each plate. The top area of the soft layer was scratched and transferred to 50 mL sterile clean tubes, and a further 1 mL of buffer was added to each plate for agar and phages washing, and then poured into the collection tubes. Tubes were left for 15 min and vortexed (Vortex-Genie-2; Inc., Bohemia, NY, USA) for 5 min, followed by centrifugation (7000× *g*) for 15–20 min at 4 °C. Pellet was discarded and the supernatant was taken, filtered, transferred into sterile clean tube, and stored at 4 °C. For the determination of each phage titer, a ten-fold serial dilution of the phage suspension was performed and counted by the overlay technique as previously described.

#### 2.2.5. Phages Host Spectrum Lytic Activity

Selection of the broadest host range phage exhibits a polyvalent behavior was performed by spot testing according to Capra, Quiberoni and Reinheimer [53]. Lytic activity of all phages was determined against 50 bacterial strains included; 27 host strains and 23 other strains were 6 of *S. aureus*, 3 of methicillin-resistant *Staphylococcus aureus* (MRSA), 2 of vancomycin-resistant *Staphylococcus aureus* (VRSA), 7 of *Bacillus cereus* and 5 of *B. subtilis*. Briefly, 10 μL of each isolated phage was spotted on each bacterial strain (1 × 10^8^ CFU/mL) in a solidified soft agar layer that had previously been poured onto TSA plates. Clear spots were examined on lawn after overnight incubation at 37 °C. In addition to the spot test, the phages host range was also confirmed by the efficiency of the plating (EOP) method according to Mirzaei and Nilsson [56] and Huang, et al. [57] with some modifications. Phages showed a wide host range were serially diluted to 1 × 10^7^ PFU/mL. In double-layer plate assays, 100 μL of phage lysate and 100 μL of overnight culture were added. The plates were then incubated according to the incubation criteria appropriate to the bacteria tested. The resulting plaques were numerated in the form of (PFU/mL) at the end of the incubation period. Experiment in triplicates were done in parallel on both tested and target hosts. The EOP was calculated by taking the average PFU on test bacteria/average PFU on lawns with the standard deviation (±SD) for triplicates. The data of EOP were interpreted in four forms, as follows: high efficiency EOP from 0.5 to 1.0; moderate efficiency EOP from 0.2 to <0.5; low efficiency EOP from 0.001 to <0.2, and inefficient EOP <0.001.

#### 2.2.6. Multiplicity of Infection (MOI) and One Single-Step Growth Tests

For all isolated phages, MOIs and single-step growth tests were performed using the previously described methods of Wang, et al. [58]. Each phage lysate (10^8^ PFU/mL) in a separate sterile tube was added to 5 mL of TSB inoculated with the original bacterial host (1.5 × 10^8^ CFU/mL) to obtain different MOIs (10, 1, 0.1, 0.01, 0.001, or 0.0001). The mixture was incubated for 4 h in shaking (220 rpm) at 37 °C. After incubation, the tubes were centrifuged at 8000× *g* for 20 min, the pellet was discarded, and the supernatant was filtered through a 0.22 mm syringe filter. The titers were then determined using the overlay method [54,55]. The MOI obtained from the highest phage titer was considered the optimal MOI of the phage.

For the determination of latency period and burst size, one-step growth test was performed as follows: 4 mL of culture host (10^7^ CFU/mL) was inoculated with 50 μL of phage lysate (10^8^ PFU/mL) at the optimal MOI. Phages were incubated in shaker at 37 °C for 5 min to adsorb, followed by centrifugation (12,000× *g*) for 1 min to remove the free phages. In 4 mL of fresh TSB medium, the pellet was suspended (time zero) and then incubated at 37 °C. One hundred μL samples were taken after 5 min of phage addition and every 10 min intervals up to 3 h of incubation period, all samples were taken for calculation of the plaques using the overlay method [54,55]. The ratio between the number of virions produced at the latency period and the number of bacterial cells initially infected is the burst size. In addition, the shortest incubation time permitting the phages production from the time between infection was defined as the latency period. 

#### 2.2.7. Transmission Electron Microscopy (TEM)

Using a phage buffer, 1 mL of high titer stock phages were washed after centrifugation (16,000× *g*) for 60 min at 4 °C. The pellet was gently suspended in 20 μL of the CM phage buffer and the supernatant was discarded. Five μL of suspended phages examined were added to the carbon grids (200 mesh), coated with formvar, and allowed to stand for 2 min. By using 2% uranyl acetate, phages were negatively stained (30 s), and excess stain was pulled off by filter paper [59]. Samples were examined by electron microscopy (Model Beckman 1010) operated at 60 KV at the Regional Center for Mycology and Biotechnology, Al-Azhar University, Cairo, Egypt [60]. 

#### 2.2.8. Thermal and pH Stability of CoNShP-3 Phage

Thermostability of CoNShP-3 phage against many temperatures (‒20 °C, 4 °C, 25 °C, 37 °C, 50 °C, 60 °C, and 75 °C) was checked after 1 h, 2 h, 4 h, 12 h, 24 h, and 1 week according to Philipson, et al. [61]. Similarly, the effect of pH levels (4, 7, 9, and 11) on the activity of CoNShP-3phage incubated at the previous times was studied according to Jamalludeen, et al. [62]. The average of phage titer was the count for each sample using the overlay method [54,55]. The experiment was performed in a triplicate with long of standard deviation (±SD).

#### 2.2.9. Degradation of CoNS Biofilms Using CoNShP-3 Phage

Biofilm removal activity of CoNShP-3 phage on the CoNS biofilms was performed in a 96-well flat-bottom polystyrene microtiter plate (Sigma-Aldrich, Costar, USA), according to Bekir, Abdallah, Ellafi and Bakhrouf [50]. The assay was against the strongest CoNS isolates (3 strains) that produced biofilms; *S. epidermidis* CFS79, *S. haemolyticus* CFS43, and *S. saprophyticus* CFS28. Briefly, each well was filled with 200 μL of culture (2 × 10^6^ CFU/mL) in a TSB medium supplemented with 0.25% glucose. Then 100 μL of CoNShP-3 phage lysate (1 × 10^6^ PFU/mL) was mixed with the tested strain(s) in some specific and marked wells (wells of anti-biofilm). Bacteria without phage treatment capable of growing, adsorbing to wells of polystyrene, and producing biofilms (wells of biofilm). Wells of TSB medium supplemented with 0.25% glucose without bacteria and phage (wells of negative control) were included. Percentages of biofilm reduction were calculated according to Else, et al. [63] and Kostaki, et al. [64] by the following equation:

[(C − B) − (T − B)]/[(C − B)] × 100(1)

where C = average OD_620 nm_ of the control group, B = average OD_620 nm_ of blank wells containing used medium and T = average OD_570 nm_ of phage-treated wells.

#### 2.2.10. Evaluation Assays of the CoNShP-3 Phage as a Natural Antibacterial

##### Determination of CoNShP-3 Phage Effect on Bacteriophage-Insensitive Mutants (BIMs)

Developed BIMs frequency for the CoNShP-3 phage was determined according to O’Flaherty, Ross, Meaney, Fitzgerald, Elbreki and Coffey [36] and O’Flynn, et al. [65]. Phage was mixed with the bacterial host culture at optimal MOI for phage infection and incubated at 37 °C for 15 min. After incubation, the obtained colonies were counted using the plate count agar method; the BIMs were calculated as (number of surviving colonies divided by the original bacterial concentration).

##### Bacteriolytic Activity of CoNShP-3 Phage Using the 96-Well Microtiter Plate

The lytic activity of CoNShP-3 phage was also confirmed by measurements of optical density (O.D_600 nm_) in a 96-well flat-bottom polystyrene microtiter plate (Sigma-Aldrich, Costar, USA), according to Anany, et al. [66] with some modifications. This test was performed on the bacterial strains, which were strongly affected by efficiency of the plating (EOP) test. Shortly the microtiter plate contains a set of positive control wells (overnight cultures of tested bacteria without phage treatment), negative control wells (TSB medium only), and test wells (phage treated bacteria). After incubation at 37 °C for 24 h in a shaker (160 rpm) with an interval of one hour, the absorbance was measured using an ELISA reader (Sunrise™-TECAN, Switzerland) with long of standard deviation (±SD) for triplicates.

##### Bacterial Challenge Assay in TSB (CoNS Culture Clearing)

Challenge assays were performed to determine the ability of CoNShP-3 phage to control CoNS strains in the broth medium. Three test flasks containing 100 mL of TSB medium have been sterilized. In two flasks, 1 mL of overnight culture (10^7^ CFU/mL) of mixture contained *S. haemolyticus* CFS43, and *S. epidermidis* CFS79 in a ratio of 1:1 was added to the TSB medium. In only one of the two flasks, 1 mL of CoNShP-3 phage suspension (10^8^ PFU/mL) was added at MOI of 0.1 (treated flask), while the other one remained without treatment (positive control flask). The third flask contained only the TSB medium (negative control flask). Three flasks were incubated in shaking condition (220 rpm) at 37 °C for 24 h. Two mL samples were taken from flasks at times 0, 1, 3, 6, 12 and 24 h, 1 mL for measurements (OD_620 nm_) using a spectrophotometer, and another 1 mL for determination of log_10_ bacterial count on TSA plates. All measurements were performed in triplicate with long of standard deviation (±SD).

##### Bacterial Challenge Assay in Food (Control CoNS Bacteria in Food)

Chicken breast was chosen as a representative sample of a food experiments, obtained from a local supermarket and transported in an icebox, and then aseptically sliced into the laboratory. The sliced chicken breast was cut into small pieces (2 cm × 2 cm square) and then 70% ethanol was used as a solution to sterilize chicken breast pieces for 3 h. After that, it was removed from the alcohol solution, washed with sterile water three times, and then allowed to dry in the Petri dish for 30 min. The microbial load was screened on TSA plates using total aerobic bacterial count according to the FDA [67]. Only those ones that are bacterial-free were used in the experiment and divided into three equal groups. 

Short Term Storage Efficacy and Stability of CoNShP-3 Phage

Previously incubated overnight cultures of *S. haemolyticus* CFS43 and *S. epidermidis* CFS79 in a ratio of 1:1 suspended in TSB was prepared. One mL of the bacterial mixture (final viable count of 6 log_10_ CFU/cm^2^) was spread into only two of the three groups previously prepared, then dried for 1 h in order to bacteria to adsorb on the chicken pieces. The other, un-inoculated (CM buffer only) group was used as a negative control (*not contaminated*, *untreated group*). 

*For CoNShP-3 Phage efficacy*; phage lysate (8 log_10_ PFU/cm^2^) at optimal MOI was added and spread over chicken breast pieces for only one of the two groups are artificially inoculated with bacteria (*contaminated*, *treated group*). It was left for 40 min to allow the phage to adsorb chicken breast pieces. While the remaining group inoculated with bacteria remains without phage treatment (*contaminated*, *untreated group*). All Petri dishes were covered with sterile stretchy plastic and stored at 4 °C for 1 week. Bacterial log_10_ reduction was enumerated on TSA plates at day 0, 1, 2, 5, and 7 of the storage periods.

*For CoNShP-3 Phage stability*; To determine the phage stability in the food over the course of the storage period, the phage titer was monitored by plaque assay [54,55] at the same experiment times as bacteria.

Long Term Storage Efficiency and Stability of CoNShP-3 Phage

The efficacy of CoNShP-3 phage for reducing CoNS in food was also assessed over the course of an entire month. Three other groups of chicken breast cuts (2 cm^2^) were prepared in the same way as mentioned above and separately placed in plates. The plates were covered in and appropriate and sterile manner, and then frozen at ‒20 °C for a month. The bacterial count (log_10_) was done at 0, 3, 7, 14, 21, and 30 days. In parallel, the stability of the CoNShP-3 phage was determined at the previous periods using the overlay technique [54,55]. 

Recovery of CoNS Bacteria from Artificially Contaminated Food

To determine the efficacy of the CoNShP-3 phage in controlling the bacteria in food, the artificially inoculated CoNS were recovered by calculating the log_10_ reduction. In a clean and sterile bag, the sample was added and mixed with one mL of PBS. Homogenized and vortexed [68] and then centrifuged (3000× *g*) for 10 min to avoid bacteriophage plating [69]. The supernatant discarded and the pellet was mixed with peptone water. The sample was serially diluted and spread onto Baird–Parker agar (Oxoid, UK) plates supplemented with egg yolk telluride emulsion, and then incubated at 37 °C for 24–48 h. The colonies (well-defined contours, grey-black, smooth, and moist colonies) were counted (CFU/mL), and then presumptive CoNS colonies sub-cultured onto Blood (7% sheep blood) Agar (BA) plates. The plates were incubated under aerobic at 37 °C for 24 h. Gram stain, coagulase, and catalase tests were performed for colonies that were produced on BA, according to Holt, Krieg, Sneath, Staley and Williams [43], Collins and Lyne [44] and Cheesbrough [45]. Gram (+ve), coagulase (−ve) and catalase (+ve) were identified as CoNS populations [70].

### 2.3. Statistical Analysis

Biofilm tests and food experiments were done in triplicates and three samples per assay were performed in each replicate. Results of thermal and pH stability of CoNShP-3 phage and efficacy in the control of CoNS in TSB medium and food were reported as triplicates with standard deviation (±SD). The data was converted to log_10_ units of the results for the phage and bacteria.

The experimental design was entirely randomized, and statistical analysis was performed using SPSS (Social Science version 26.00) statistical software at a probability level of 0.05. Quantitative analyses were obtained using one-way ANOVA with least significant difference (LSD) test variance analysis with the parametric distribution of Levene’s study. The interval of confidence was set at 95%, and the agreed error margin was set at 5%. Graphs were drawn with GraphPad Prism 8.

## 3. Results

### 3.1. Characteristics of Bacterial Isolates

#### 3.1.1. CoNS Isolates and Antibiotics Susceptibility Profile 

Results of the primary identification of presumptive CoNS isolates, previously isolated from samples of cheese and meat products, showed that only 93 isolates represent the CoNS populations, while the others were not and were ignored. The antibiotic-resistance profiles of CoNS showed that 27 (29%) of 93 isolates displayed a multidrug-resistant (MDR) behavior. All 27 MDR-CoNS isolates were resistant (according to the CLSI guidelines) to at least 7 different antibiotics from the 16 experimentally tested. The resistance was found towards ampicillin, oxacillin, erythromycin, gentamicin, clindamycin, tetracycline, and ciprofloxacin with bacterial resistance 100%. The results of Biomerieux VITEK 2 system identification for isolates showed that, the most isolated strains were *Staphylococcus haemolyticus* (9; 33.3%), *Staphylococcus saprophyticus* (7; 25.9%), *Staphylococcus epidermidis* (7; 25.9%), and *Staphylococcus hominis* (4; 14.8%).

#### 3.1.2. Biofilm Formation Activity of MDR-CoNS Strains

**Figure 1:**
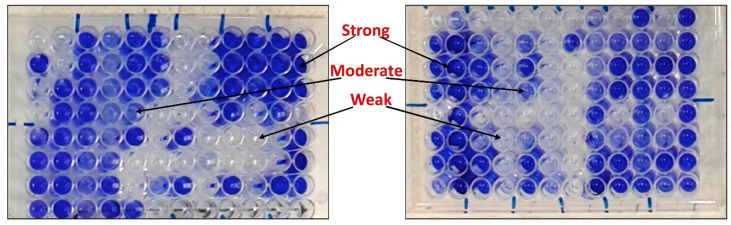
Microtiter ELISA plate shows the patterns of biofilm formation by MDR-CoNS strains. The strains listed in Table 1 were cultured overnight in in a 96-well flat-bottom polystyrene microtiter plate in trypticase soy broth (TSB) medium supplemented with 0.25% glucose. The cells that adhered to the plate after washing with phosphate-buffered saline (PBS) were then visualized by staining with crystal violet and solubilized in 1% *w*/*v* SDS. The optical density was determined at optical density 570 nm (OD_570 nm_) and the assay was performed in triplicate a long with the standard deviation (±SD).

The results obtained in Table 1 show that MDR-CoNS strains have a different ability to produce biofilm (Figure 1). Thirteen isolates (13/27; 48%) show a strong production of the biofilm; 5 isolates of *S. haemolyticus*, 2 of *S. saprophyticus* and 6 of *S. epidermidis*, while *S. hominis* strains did not produce a strong biofilm (Table 1). Moderate biofilm was produced by 6 (22.2%) isolates; 4 isolates of *S. haemolyticus and S. saprophyticus* two each and 2 of *S. epidermidis* and *S. hominis*, one each (Table 1). In contrast, the weak biofilm was produced by 8 (29.6%) isolates; 2 isolates of *S. haemolyticus*, 3 of *S. saprophyticus*, 1 of *S. epidermidis* and 3 of *S. hominis* (Table 1). It was worth observed that, the strongest isolates (8/13; 61.5%) producing biofilm were sourced from cheese samples (Table 1).

### 3.2. CoNS Bacteriophages

#### 3.2.1. Isolation and Characterization

**Figure 2:**
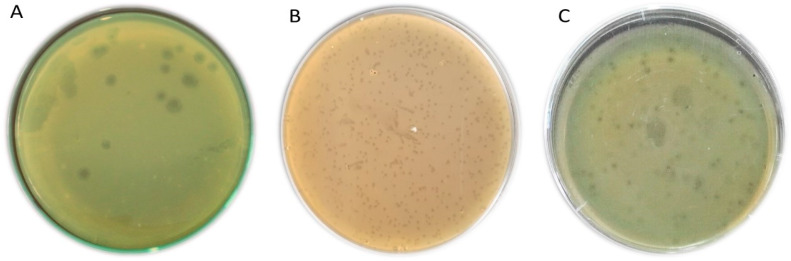
Plaques of different CoNS Bacteriophages. (**A**) CoNShP-3, (**B**) CoNSsP-1, and (**C**) CoNSeP-2.

Of the 30 different sewage samples that were examined to isolate CoNS bacteriophages, 11 samples displayed positive results for the presence of phages using spotting assay. The overlay technique showed the formation of either turbid or clear plaques on the plates of *S. haemolyticus*, *S. saprophyticus*, and *S. epidermidis*, while *S. hominis* did not show positive results for the phages. A total of 29 different CoNS phages were isolated, but only phages (6 out of 29) produce clear lytic plaques were purified and propagated (the phages titers were 10^8^ to 10^11^ PFU/mL). The plaques morphology of CoNS phages was clear, regular circular, and irregular in shape with a diameter of about 0.4–3 mm (Figure 2). The selected bacteriophages in this study were; three isolates for *S. haemolyticus* named CoNShP-1, CoNShP-2, and CoNShP-3, one isolate for *S. saprophyticus* named CoNSsP-1 and two isolates for *S. epidermidis* named CoNSeP-1 and CoNSeP-2.

#### 3.2.2. Host Range Spectrum and EOP 

A collection of 50 different bacterial strains that contained the lawns strains (27 MDR-CoNS isolates) used to isolate CoNS phages, and 23 of other strains that were used to determine the host range spectrum and efficiency of plating (EOP) of CoNS phages (Table 2). The lytic activity of CoNShP-3 was the broadest spectrum, as it was able to lyse 32 (64%) out of 50 tested bacteria. It is extremely important to find that CoNShP-3 phage has exhibited a typical polyvalent behavior, where it was able to infect the CoNS and coagulase-positive staphylococci (CoPS) that included *S. aureus*, methicillin-resistant *S. aureus* (MRSA) and vancomycin-resistant *S. aureus* (VRSA) strains as well as, both *B. cereus* and *B. subtilis* strains. Furthermore, CoNShP-3 polyvalent phage showed a high EOP in 17 out of 50 bacterial strains. Although CoNSeP-2 phage was ranked second (19/50; 38%) in extent of the host range, it did not show a polyvalent behavior and exhibited a high EOP in 9 out of 50 strains. CoNShP-1, CoNSsP-1, CoNSeP-1, and CoNShP-2 did not show an effect on the CoPS, *B. cereus* and *B. subtilis* strains tested with no EOP. The corresponding host ranges were 36% (18/50), 32% (16/50), 30% (15/50), and 26% (13/50), respectively, and mostly they were moderately in EOP against CoNS strains.

#### 3.2.3. Single-Step Growth Curve and Multiplicity of Infection (MOIs)

**Figure 3:**
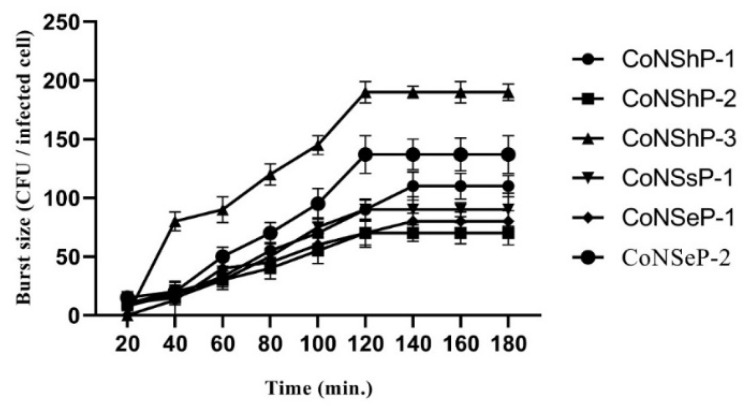
Single-step growth curve of the six CoNS isolated phages (CoNShP-1, CoNShP-2, CoNShP-3, CoNSsP-1, CoNSeP-1, and CoNSeP-2); values of burst size and latency period were represented vertically and horizontally, respectively.

To all CoNS phages, latency periods and burst sizes were determined using single-step growth curve (Figure 3). The latency periods of the phages were 20–120 min, and the burst sizes were 110, 70, 190, 90, 80 and 137 PFU/infected cell for CoNShP-1, CoNShP-2, CoNShP-3, CoNSsP-1, CoNSeP-1, and CoNSeP-2, respectively. Both CoNShP-3 and CoNSeP-2 exhibited a higher burst size, but CoNShP-3 is most likely due to the larger number of phage progeny released. In addition, the optimal MOIs for the previous phages were 0.01, 0.001, 0.01, 0.01, 0.01, and 0.01, respectively.

#### 3.2.4. Morphology (Electron Microscopy)

**Figure 4:**
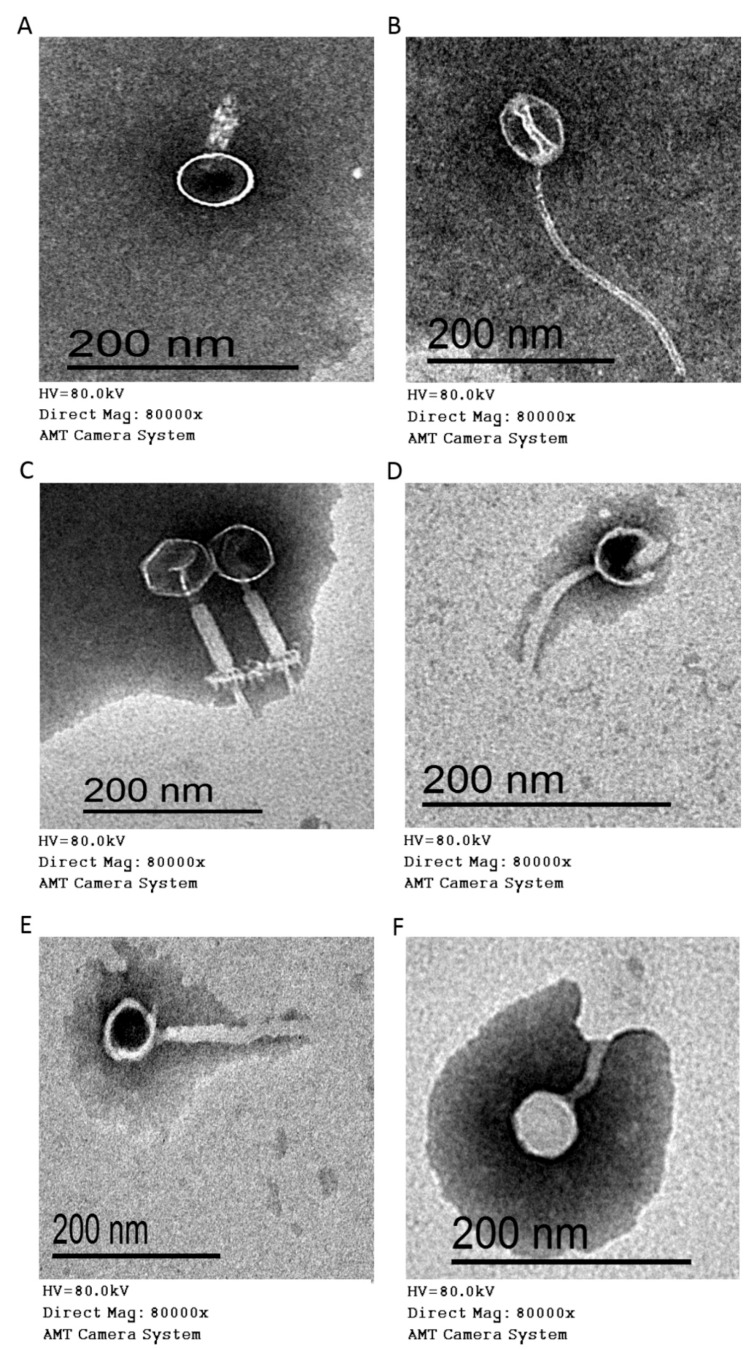
Electron micrographs at 80,000× of (**A**) CoNShP-1 phage, (**B**) CoNShP-2 phage, (**C**) CoNShP-3 phage, (**D**) CoNSsP-1 phage, (**E**) CoNSeP-1 phage and (**F**) CoNSeP-2 phage. Bar 200 nm.

The morphological features of the CoNS phages were examined to determine the morphotype specific groups to which the phages belonged (Figure 4), according to the system (ICTV) used in the classification of the viruses. The six isolated phages were classified into 2 morphotypes (*Myoviridae* and *Siphoviridae* families) of the order *Caudovirales* (had tails). The myophages included CoNShP-1, CoNShP-3, and CoNSeP-2 isolates, contained a contractile tails (Figure 4A,C,F), with characteristic baseplate and bulbous terminal spikes for CoNShP-3 phage (Figure 4C). The head features were hexagonal in shape with icosahedral symmetry for CoNShP-3 and CoNSeP-2 (Figure 4C,F), while CoNShP-1 had a round head shape with isometric symmetry (Figure 4A). On the other hand, the siphophages included CoNShP-2, CoNSsP-1, and CoNSeP-1, were distinguished by a long non-contractile tail without baseplate. The features of the head were round in shape for CoNSsP-1 and CoNSeP-1, while CoNShP-2 showed icosahedral head (Figure 4B,D,E). To all phages, the tail lengths and the head diameters presented in Table 3.

#### 3.2.5. Thermal and pH Stability of CoNShP-3 Phage

**Figure 5:**
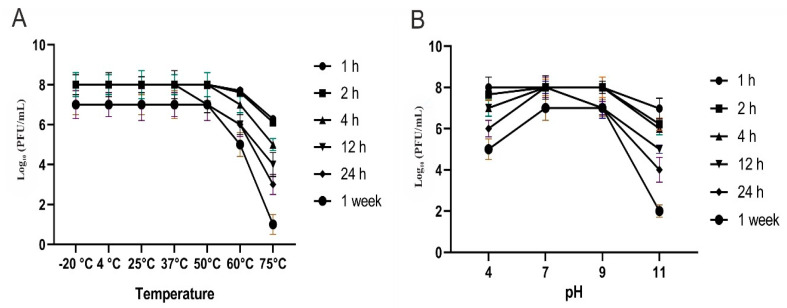
Titers stability of CoNShP-3 phage at different time periods (2 h, 4 h, 12 h, 24 h, and 1 week). (**A**) under different temperatures. (**B**) under different pH values.

Figure 5A shows that CoNShP-3 phage was stable at temperatures −20 °C, 4 °C, 25 °C, 37 °C and 50 °C without insignificant (*p* < 0.05) titer reduction at all-time intervals. At 60 °C, a minor titer reduction around 1.96 ± 0.36 and 2.12 ± 0.21 log_10_ PFU/mL was observed at 24 h and a week, respectively. Strongest titer reduction (significant) was found at 75 °C by 3.89 ± 0.33 and 4.21 ± 0.40 log_10_ PFU/mL at 12 and 24 h, respectively, while at a week of incubation, CoNShP-3 phage lost most of the titer activity around 6.02 ± 0.44 log_10_ PFU/mL.

In context, Figure 5B indicates that CoNShP-3 phage was extremely stable in a wide range of pH, and the phage did not lose its infectivity. In a neutral condition, the phage titer remained stable throughout the different measurement periods. Indeed, CoNShP-3 phage was more stable in acidic conditions than alkaline conditions (pH = 11), where at pH of 4, a reduction of log_10_ count was about 0.97 ± 0.11, 1.67 ± 0.11, and 2.34 ± 0.23 PFU/mL at 12, 24 h and a week, respectively. On the other hand, at alkaline pH of 11, the phage activity was reduced by a log_10_ of 2.78 ± 0.21 and 3.09 ± 0.23 PFU/mL at 12 and 24 h, respectively, while a significant (*p* < 0.05) log_10_ reduction was observed by 5.53 ± 0.29 PFU/mL at a week of treatment.

#### 3.2.6. Anti-Biofilm Activity of CoNShP-3 Phage Against CoNS Strains

**Figure 6:**
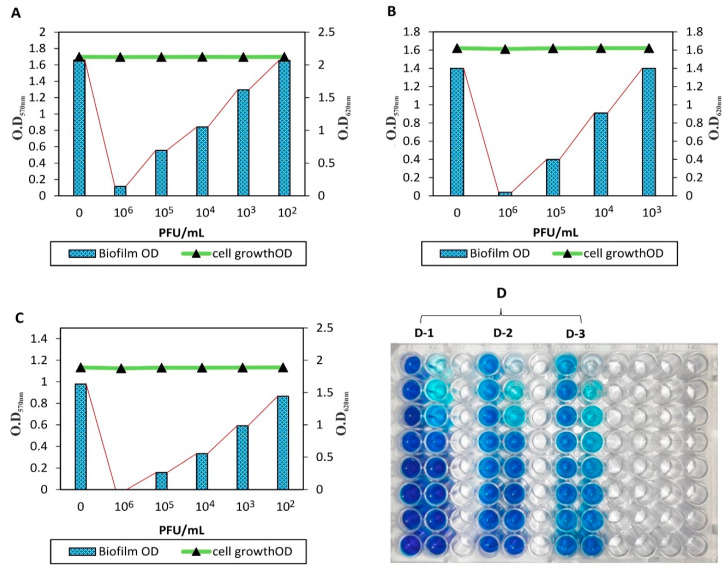
Qualitative anti-biofilm activity of CoNShP-3 phage in 96-well microplate. (**A**) *S. epidermidis* CFS79 biofilms. (**B**) *S. haemolyticus* CFS43 biofilms. (**C**) *S. saprophyticus* CFS28 biofilms. (**D**) 96-tissue culture plate [D-1, wells of biofilm control (left), and wells of anti-biofilm (right) of *S. epidermidis* CFS79; D-2, wells of biofilm control (left), and wells of anti-biofilm (right) of *S. haemolyticus* CFS43; D-3, wells of biofilm control (left), and wells of anti-biofilm (right) of *S. saprophyticus* CFS28].

The efficacy of CoNShP-3 phage against biofilm of *S. epidermidis* CFS79, *S. haemolyticus* CFS43, and *S. saprophyticus* CFS28 was evaluated using the method of tissue culture plate (Figure 6D). *S. epidermidis* CFS79 biofilm (1.657 OD_570 nm_) was reduced by 93% (0.116 OD_570 nm_), 66.4% (0.556 OD_570 nm_), and 49% (0.841 OD_570 nm_) using CoNShP-3 phage at 10^6^, 10^5^, and 10^4^ PFU/mL, respectively, with no effect on bacterial cell growth (2.122 OD_620 nm_) (Figure 6A). *S. haemolyticus* CFS43 biofilm (1.401 OD_570 nm_) was also disrupted by 97% (0.038 OD_570 nm_), 71.5% (0.399 OD_570 nm_), and 35% (0.91 OD_570 nm_) at 10^6^, 10^5^, and 10^4^ PFU/mL of CoNShP-3 phage titer, respectively, with no variation in cell growth (1.621 OD_620 nm_) (Figure 6B).

On the other hand, *S. saprophyticus* CFS28 biofilm (0.979 OD_570 nm_) was completely removed by 100% (0.000 OD_570 nm_) at 10^6^ PFU/mL. While at 10^5^ and 10^4^ PFU/mL, the biofilm was reduced by 83.7% (0.159 OD_570 nm_) and 66% (0.332 OD_570 nm_), respectively, and the cell growth remained stable (1.889 OD_620 nm_) (Figure 6C).

#### 3.2.7. Efficacy and Stability of the CoNShP-3 Phage as a Natural Antibacterial

##### Lytic Activity of CoNShP-3 Phage Using 96-Well Microtiter Plate

To confirm the bacteriolytic effect of CoNShP-3 phage, the lytic activity was assayed spectrophotometry (OD_620 nm_) in 96-well microtiter plate against the 17 bacterial strains that showed high EOP as shown in Table 2. It was found that along the entire incubation period (24 h), positive control wells show an increase in the absorbance continuously, while optical densities of negative control wells remained unchanged. On the other hand, wells of bacterial culture containing CoNShP-3 showed low increase in absorbance until the first hour of incubation. Followed by a complete inhibition of bacterial growth, which was measured every hour during the incubation period; this indicates that all 17 bacterial strains were lysed by the CoNShP-3 phage as a result of its strong lytic activity. 

##### Control of CoNS Strains in TSB Medium (Culture Clearing) and BIMs Formation

**Figure 7:**
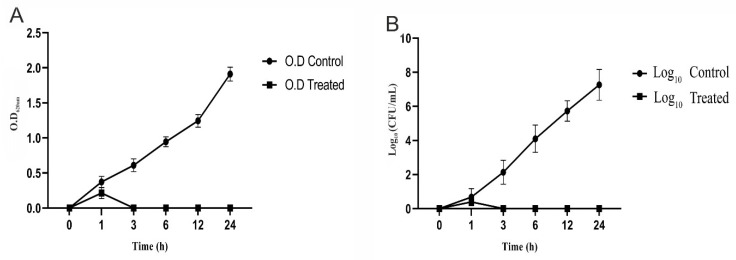
Challenge of a CoNS culture (*S. haemolyticus* CFS43 and *S. epidermidis* CFS79) with CoNShP-3 phage at 37 °C. (**A**) in broth medium of TSB (measurements of OD_620 nm_), and (**B**) in the solid medium of TSA (measurements of colonies count CFU/mL).

To determine CoNShP-3 phage potency in clearing the bacterial culture in broth medium (TSB), challenge experiment against a mixture of *S. haemolyticus* CFS43 and *S. epidermidis* CFS79 culture in a ratio of 1:1 was performed. Within one-hour, CoNShP-3 phage began to inhibit bacterial growth, while at 3 h of the experiment the culture was reduced to an undetectable range. In contrast, the absorbance of the positive control culture increased to 1.911 ± 0.21 OD_620 nm_ at the end of the 24 h incubation period (Figure 7A). Indeed, plate counts as a confirmed test assay showed no bacterial colonies from 3–24 h of incubation, whereas at the end of the experiment, the count significantly (*p* < 0.05) reduced from 7.24 ± 0.47 log_10_ CFU/mL to undetected colonies (Figure 7B). It is worth noting that, after 3 h of treatment with CoNShP-3 phage until the end of the experiment, no BIMs were formed as a result of the absence of viable bacterial count on TSA plates.

##### Control of CoNS Strains in Food

The efficacy of CoNShP-3 phage in biocontrol against artificially inoculated CoNS bacteria to chicken breast sections, in addition to the phage stability were determined at short and long storage periods.

**Figure 8:**
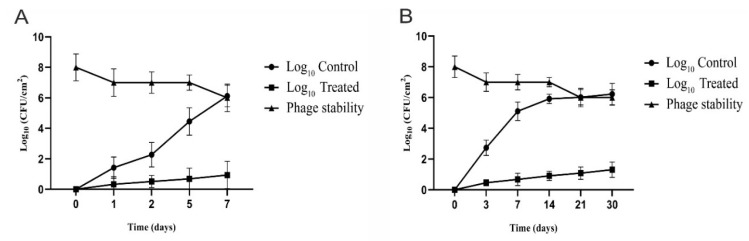
Biocontrol efficacy and stability of CoNShP-3 phage in artificially inoculated food by CoNS bacteria (*S. haemolyticus* CFS43 and *S. epidermidis* CFS79). (**A**) Short term storage and stability (7 days) at 4 °C. (**B**) Long term storage and stability (1 month) at −20 °C.

Short Term Storage and Stability: Figure 8A shows that the log_10_ bacterial count has significantly (*p* < 0.05) increased in untreated (positive control) chicken breast sections stored at 4 °C for a period of 7 days. Recoverable log_10_ of CoNS bacteria at day 2 and 7 it was 2.27 ± 0.22 and 6.12 ± 0.36 log_10_ CFU/cm^2^, respectively. A significant (*p* < 0.05) reduction in log_10_ count was observed in treated chicken breast sections by 5.18 ± 034 log_10_ CFU/cm^2^, at day 7 with MOI of 10,000. Evidently, the CoNShP-3 phage initial titer (8 log_10_ PFU/cm^2^) show insignificant decrease by 1.33 ± log_10_ PFU/cm^2^ until day 7 and relatively remained stable with the recoverable titer was 6.67 ± 0.45 PFU/cm^2^.

Long Term Storage and Stability: Figure 8B indicates the efficacy and stability of the CoNShP-3 phage to reduce CoNS bacteria in a long-term manner at −20 °C for one month. At day 3 and 30 the log_10_ viable counts of CoNS bacteria was 2.74 ± 0.21 and 6.23 ± 0.51 CFU/cm^2^, respectively, while at the same two periods the CoNShP-3 phage caused a sharp decrease (significant reduction) in the log_10_ counts to 0.46 ± 0.11 and 1.31 ± 0.31 CFU/cm^2^, respectively. In addition to, the phage titer recovered was simply affected (insignificant reduction) by 1.92 ± 0.11 log_10_ PFU/cm^2^ out of initial titer (8 log_10_ PFU/cm^2^), this indicates the stability of the phage and its efficiency in controlling CoNS bacteria in food, which makes it a promising candidate.

## 4. Discussion

Natural and safe antimicrobials to control foodborne bacteria are of paramount importance as they do not adversely affect food quality or human health [16,18,71]. The traditional view of coagulase-negative staphylococci (CoNS) spp. as a non-pathogen has changed, as it is now considered to be the main cause of human and veterinary infections in many countries [5,72]. The enterotoxigenic influence of CoNS bacteria in food intoxication has also been detected and increased [4]. In addition, CoNS can produce a biofilm that is resistant to the effects of antibiotics, detergents and disinfectants so that it retains its survival and ability to infect [9,10,73]. Many foods are frequently associated with CoNS, including dairy products, meat and meat products and many other foods [74].

In this study, 93 out of 137 isolates from samples of white cheese and meat products were identified as CoNS populations. Antibiogram profiles of these isolates showed that 37 CoNS were multi-drug resistant (MDR) strains. These strains included *Staphylococcus haemolyticus* (n = 9/27), *Staphylococcus saprophyticus* (n = 7/27), *Staphylococcus epidermidis* (n = 7/27), *Staphylococcus hominis* (n = 4/27). Consistent with our results, Kırkan, et al. [75] found that 20% of samples examined had CoNS strains, including *S. saprophyticus*, *S. haemolyticus*, *S. epidermidis*, *S. xylosus*, *S. hominis* and other strains. In addition, CoNS were isolated from dairy products at different concentrations by Ruaro, et al. [76]. CoNS were also isolated from samples of lamb meat and beef in another study by Guran and Kahya [77]. Moreover, studies conducted by Pamuk, Eker and Yıldırım [40] and Kenar, et al. [78] isolated the CoNS from food with a high antibiotic resistance behavior, in particular to penicillin. Resistance in the CoNS may be due to the fact that the species carry genes (e.g., *mecA* gene) that work to combat the action of many antimicrobials [79]. The *mecA* gene is located next to the chromosomal replication site; therefore, it can be easily be transferred between the CoNS [80].

In the current study, of the 27 MDR-CoNS, 13 isolates with a strong biofilm production capability, with three isolates (*S. epidermidis* CFS79, *S. haemolyticus* CFS43, and *S. saprophyticus* CFS28) of different strains, are more potent in production. Similar findings have been reported by Foka, Chini, Petinaki, Kolonitsiou, Anastassiou, Dimitracopoulos and Spiliopoulou [11] that strains of MDR-*S. epidermidis* and *S. haemolyticus* are capable of colonization and associated with neonate disease through the production of biofilms. The mechanism of biofilm formation in CoNS strains is controlled by the Quorum Sensing (QS) *agr* system through the production of ClpP protease [81]. ClpP protease involved in biofilm formation and other virulence factors via lysis of misfolded proteins. Other proteins that are also involved in the formation of biofilm are called biofilm-associated protein (Bap) [82]. Bap mediates the growth [83], attachment, and biofilm accumulation phase [82].

Interestingly, a total of 29 (6 lytic phages were selected) different phages were isolated in this study using overlay technique, in order to measure their efficacy as a natural antibacterial against isolated MDR-CoNS pathogens. Likewise, to isolate bacteriophages from environmental samples, Jensen, et al. [84] and Xu, et al. [85] isolated the phage from raw fecal matter, Mirzaei and Nilsson [56] and Yu, Mathieu, Li, Dai and Alvarez [37] from sewage samples, Uchiyama, et al. [86] and Lin, et al. [87] from water samples, Anand, et al. [88] from soil samples. As well as in line with our findings, the previous study by Deghorain, et al. [89] isolating coagulase-negative staphylococci phages against *S. hominis* and *S. capitis* species.

It is worth mentioning that the FDA has approved the use of phages as food preservatives against foodborne bacteria [90] as well as in the treatment of bacterial infection [91]. In the current study, six isolated phages showed a different spectrum of the host range and EOP activity. The narrow or wide range of bacteriophages may be due to the variation in the phage recognition site and bacterial membrane receptors (blocking adsorption site) as well as modification of restriction endonuclease system [92].

Interestingly, the six isolated phages were lytic against many CoNS strains, and in particular, CoNShP-3 showed typical polyvalent behavior with high EOP lytic activity. Previous study reported that phages could infect and therefore destroy a variety of *Staphylococcus* strains such as *S. saprophyticus*, *S. intermedius*, and other *Staphylococcus* species [93]. Phages with a wide host spectrum are more useful for controlling many different species of bacteria that cause infections [94,95]. Previously, it was found that SEP1 CoNS phage has a broad host range capable of infecting all tested *S. epidermidis* strains of different origin [96]. Although phages are specialized for their hosts in infection based on bacterial receptors and other factors [97], other phages are polyvalent (i.e., not host-specific) and have the ability to infect many bacteria of different genera [36,37,38,39]. This behavior may be due to a common recognition sites of bacterial receptors or to a close phylogenetic link between similar bacteria [98]. The bacteriolytic activity of the Gram-positive phages is attributed to the production of endolysins with dual action domains (enzymatically active domains (EADs) and a cell wall binding domain (CBD) of peptidoglycan (PG) hydrolysis [99]. These domains involved in the lysis of infected bacteria by activation of endopeptidase and amidase during infection [100]. In the same context, the bacteriolytic activity of the Gram-positive phages is attributed also to the presence of PG_binding_1 proteins that are specific to the endolysins of these phages [101].

In the present study, phages latency periods were approximately 20–120 min, with respect to MOIs that CoNShP-3 and CoNShP-2 had higher burst size, while CoNShP-3 showed the highest number of phage progeny. The burst size and latency period of phages are key factors for their selection in biocontrol, which plays a vital role in the destruction of the bacterial host [102]. According to our study, many previous studies have confirmed that phages with short latent period are most needed due to the high-processivity DNA polymerase and DNA polymerases homology of these phages [103]. Indeed, to achieve a high level of reduction for pathogens, phages should generally be used at high concentrations [104,105,106]. In addition, the cytoplasmic membrane of bacterial cells may undergo hydrolysis without replication by phage adsorption at a high titer level [107,108]. 

The six CoNS phages isolated in this study were members of the *Myoviridae* and *Siphoviridae* families involved in the *Caudovirales* order. The microscopic characteristics of these phages were found to be similar to those specified for other phages in the study by Klumpp, et al. [109] and Łobocka, et al. [110] which belonged to previous families. Furthermore, our results were consistent with another study reported that tailed phages belong to the order of *Caudovirales*, which includes the families of *Myoviridae*, *Siphoviridae*, and *Podoviridae* [111].

The results of this study showed that CoNShP-3 phage had a strong lytic activity, and the results of the bacterial challenge experiments confirmed this. Within a one-hour period, CoNShP-3 phage inhibits bacterial growth while undetectable growth was achieved at 3 h of the assay. In line with these findings but with another bacterial host, the *E. coli* O104:H4 phage can inhibit the host in growth medium for 24 h at 37 °C [112].

Noteworthy, CoNShP-3 phage exhibited thermostability at different temperatures −20 °C, 4 °C, 25 °C, 37 °C, and 50 °C, although 60 °C had a simple effect on CoNShP-3 titer, 75 °C had a dramatic decrease in phage titer. Previous studies have reported that temperature is a critical point in survival, attachment ability, the time of the latent period, occurrence, and viability [33]. As a result, we can mostly say that myophages and siphophages are considered to be heat-tolerant phages [32]. Similarly, the phage titer count remained constant at a wide pH range (4, 7, and 9) and the phage did not lose its infectivity. However, CoNShP-3 phage was more stable under acidic conditions than under alkaline conditions. Consistent with our results, the previous study reported that at pH ranged from 5.0 to 9.0; most bacteriophages remained stable [34]. It was also found that pH could affect the aggregation of the bacteriophages; for instance, MS2 phage at a pH of less than or equal to isoelectric point (*p*I = 3.9) showed a strong ability to aggregate [113].

In this study, CoNShP-3 phage showed a remarkable effect against tested CoNS biofilms. Both *S. epidermidis* CFS79 and *S. haemolyticus* CFS43 biofilms decreased by 93% and 97%, respectively, at 106 PFU/mL, compared with *S. saprophyticus* CFS28 biofilm was removed by 100%. Consistent studies in the same line have been reviewed to confirm our results, which have shown that bacterial biofilms are sensitive to phage-mediated remove in vitro assays [27,28,30,31,114]. Phages are being developed to attack biofilm-embedded cells that are widely spread throughout their ecosystems [92]. The high cell density of the bacterial biofilm enables virions to spread rapidly across the compacted neighboring cells [92]. The mechanism by which phages can remove biofilms, phage virions penetrate EPS layers of biofilms; they becomes less complicated [115], less density [116,117] or no longer thick [118]. In addition, phage-biofilm interactions may be caused by the degradation of the biofilm matrix by the production of EPS depolymerases, which are phage-encoded enzymes [29,119,120]. Since there is a strong fit between the enzyme and the EPS structure, these EPS depolymerases lead to the degradation of the biofilm structure [120].

Finally, in the present study, CoNShP-3 phage showed a high efficacy in the reduction of artificially inoculated CoNS strains in chicken breast sections and was stored for short and long periods of time. The chicken breast sections treated with phages showed a strong decrease in log_10_ count at 7 day, with log_10_ reductions of 84.6% CFU/cm^2^. Moreover, the phage titer remained infective (small losses in titers). Likewise, long-term storage efficacy and phage stability have shown promising results. After a full month of storage, the CoNShP-3 phage showed an impressive reduction of 79% log_10_ CFU/cm^2^ with a high recoverable virion stability. Other studies are consistent with our results in the use of phage as a biocontrol agent in the same type of food matrix but against another targeted bacterium [121,122].

## 5. Conclusions

The results of this study have shown that CoNS strains isolated from food have demonstrated resistance to many antibiotics; therefore, are no longer working with traditional antibacterials. This strongly encourages the use of bacteriophage as a natural alternative biocontrol agent.

Based on our findings of polyvalent lytic phages, for example, CoNShP-3 phage is a promising candidate for control of CoNS strains and their biofilms in food, in coordination with other researchers. These studies have shown that phages improve food quality and safety by controlling pathogenic bacteria in food and their biofilms [22,105].

## Figures and Tables

**Table 1:** Biofilm-forming capacity of 27 multi-drug resistant-coagulase-negative staphylococci (MDR-CoNS) strains by tissue culture plate method (TCP).

MDR-CoNS Strains	Source	Growth at optical density 620 nm (OD_620 nm_)	Biofilm at optical density 570 nm (OD_570 nm_)	Biofilm Production
*S. haemolyticus* CFS14	Cheese	1.570	1.314	Strong
*S. haemolyticus* CFS22	Cheese	1.551	1.311	Strong
*S. haemolyticus* CFS29	Cheese	1.131	0.470	Moderate
*S. haemolyticus* CFS36	Cheese	1.462	1.274	Strong
*S. haemolyticus* MFS11	Meat	0.960	0.136	Weak
*S. haemolyticus* MFS19	Meat	1.244	0.388	Moderate
*S. haemolyticus* CFS43	Cheese	1.621	1.401	Strong
*S. haemolyticus* MFS53	Meat	1.611	1.344	Strong
*S. haemolyticus* MFS61	Meat	0.883	0.119	Weak
*S. saprophyticus* CFS6	Cheese	0.844	0.199	Weak
*S. saprophyticus* CFS9	Cheese	1.008	0.511	Moderate
*S. saprophyticus* CFS28	Cheese	1.889	0.979	Strong
*S. saprophyticus* CFS47	Cheese	0.907	0.200	Weak
*S. saprophyticus* CFS48	Cheese	0.901	0.188	Weak
*S. saprophyticus* CFS55	Cheese	1.749	0.877	Strong
*S. saprophyticus* MFS17	Meat	0.969	0.439	Moderate
*S. epidermidis* CFS2	Cheese	2.071	1.570	Strong
*S. epidermidis* CFS10	Cheese	1.884	1.368	Strong
*S. epidermidis* CFS73	Cheese	1.095	0.320	Moderate
*S. epidermidis* CFS79	Cheese	2.122	1.657	Strong
*S. epidermidis* MFS3	Meat	1.971	1.441	Strong
*S. epidermidis* MFS7	Meat	1.714	1.504	Strong
*S. epidermidis* MFS45	Meat	2.100	1.542	Strong
*S. hominis* CFS2	Cheese	1.001	0.170	Weak
*S. hominis* CFS34	Cheese	0.942	0.134	Weak
*S. hominis* MFS48	Meat	1.245	0.401	Moderate
*S. hominis* MFS51	Meat	1.081	0.199	Weak

**Table 2:** Host Range and EOP Patterns of CoNS Isolated Bacteriophages.

Bacteria	CoNS Phages Isolates											
	CoNShP-1		CoNShP-2		CoNShP-3		CoNSsP-1		CoNSeP-1		CoNSeP-2	
	ST	EOP	ST	EOP	ST	EOP	ST	EOP	ST	EOP	ST	EOP
*S. haemolyticus* CFS14	−	L	−	L	+	H	+	M	−	L	−	L
*S. haemolyticus* CFS22	+	M	−	L	+	H	+	M	−	L	+	H
*S. haemolyticus* CFS29	+	H	+	M	+	H	−	L	−	L	+	M
*S. haemolyticus* CFS36	+	H	+	M	+	H	−	L	+	M	+	M
*S. haemolyticus* MFS11	−	L	−	L	+	H	−	L	−	L	+	M
*S. haemolyticus* MFS19	+	M	+	M	+	H	−	L	+	M	−	L
*S. haemolyticus* MFS43	+	H	+	H	+	H	+	M	+	M	+	H
*S. haemolyticus* MFS53	+	H	+	H	+	H	+	M	+	M	−	L
*S. haemolyticus* MFS61	+	H	−	L	+	H	+	M	+	M	+	M
*S. saprophyticus* CFS6	−	L	−	L	+	H	+	H	−	L	−	L
*S. saprophyticus* CFS9	−	L	−	L	−	L	+	H	−	L	−	L
*S. saprophyticus* CFS28	+	M	−	L	+	M	+	H	−	L	+	M
*S. saprophyticus* CFS47	+	M	+	M	+	M	+	H	+	M	+	M
*S. saprophyticus* CFS48	+	M	+	M	+	M	+	H	+	M	+	M
*S. saprophyticus* CFS55	+	M	+	M	+	M	+	H	+	M	+	M
*S. saprophyticus* MFS17	+	M	+	M	+	M	+	H	−	L	−	L
*S. epidermidis* CFS2	−	L	−	L	+	M	+	M	+	M	+	H
*S. epidermidis* CFS10	+	M	−	L	+	M	−	L	+	M	+	H
*S. epidermidis* CFS73	+	H	+	M	+	H	−	L	−	L	+	H
*S. epidermidis* CFS79	+	M	+	M	+	M	−	L	+	M	+	H
*S. epidermidis* MFS3	−	L	−	L	+	M	+	M	+	H	+	H
*S. epidermidis* MFS7	+	H	+	M	+	H	+	M	+	H	+	H
*S. epidermidis* MFS45	+	M	−	L	+	H	−	L	+	M	+	H
*S. hominis* CFS2	−	L	−	L	−	N	+	M	_	L	−	L
*S. hominis* CFS34	+	M	+	M	+	H	−	L	+	M	+	M
*S. hominis* MFS48	−	L	−	L	−	L	−	L	_	L	−	L
*S. hominis* MFS51	−	L	−	L	+	H	−	L	_	L	+	M
*S. aureus* 1	−	N	−	N	−	L	−	N	−	N	−	N
*S. aureus* 2	−	N	−	N	−	L	−	N	−	N	−	N
*S. aureus* 3	−	N	−	N	+	M	−	N	−	N	−	N
*S. aureus* 4	−	N	−	N	+	M	−	N	−	N	−	N
*S. aureus* 5	−	N	−	N	−	N	−	N	−	N	−	N
*S. aureus* 6	−	N	−	N	+	H	−	N	−	N	−	N
MRSA 1	−	N	−	N	−	L	−	N	−	N	−	N
MRSA 2	−	N	−	N	+	M	−	N	−	N	−	N
MRSA 3	−	N	−	N	−	L	−	N	−	N	−	N
VRSA 1	−	N	−	N	−	L	−	N	−	N	−	N
VRSA 2	−	N	−	N	+	H	−	N	−	N	−	N
*B. cereus* 1	−	N	−	N	−	N	−	N	−	N	−	N
*B. cereus* 2	−	N	−	N	−	N	−	N	−	N	−	N
*B. cereus* 3	−	N	−	N	−	N	−	N	−	N	−	N
*B. cereus* 4	−	N	−	N	−	N	−	N	−	N	−	N
*B. cereus* 5	−	N	−	N	+	M	−	N	−	N	−	N
*B. cereus* 6	−	N	−	N	−	N	−	N	−	N	−	N
*B. cereus* 7	−	N	−	N	−	N	−	N	−	N	−	N
*B. subtilis* 1	−	N	−	N	−	N	−	N	−	N	−	N
*B. subtilis* 2	−	N	−	N	+	M	−	N	−	N	−	N
*B. subtilis* 3	−	N	−	N	+	M	−	N	−	N	−	N
*B. subtilis* 4	−	N	−	N	−	N	−	N	−	N	−	N
*B. subtilis* 5	−	N	−	N	−	N	−	N	−	N	−	N

ST: spot test, +: positive spot test (strain is susceptible to the phage), −: negative spot test (strain is not susceptible to the phage), MRSA: methicillin-resistant *S. aureus*, VRSA: vancomycin-resistant *S. aureus*, EOP; efficiency of plating, H: high EOP from 0.5–1.0, M: moderate EOP from 0.2–0.4, L: low EOP from 0.001–0.1, N: no EOP (inefficient) <0.001.

**Table 3:** Morphotypes and dimensions of the CoNS isolated phages.

Isolate Character	Tail Length (nm)	Head Diameter (nm)
**Myophages**		
CoNShP-1	61.59	67.59
CoNShP-3	152.16	64.12
CoNSeP-2	79.13	48.96
**Siphophages**		
CoNShP-2	263	54.38
CoNSsP-1	95.42	53.67
CoNSeP-1	176.37	46.98

## References

[1] Havelaar A., Kirk M.D., Torgerson P., Gibb H.J., Hald T., Lake R.J., Praet N., Bellinger D.C., de Silva N.R., Gargouri N. (2015). World Health Organization Global Estimates and Regional Comparisons of the Burden of Foodborne Disease in 2010. PLoS Med..

[2] Matthews K.R., Kniel K.E., Montville T.J. (2017). Food Microbiology: An Introduction.

[3] (2006). US food and drug (FDA/CFSAN) bad bug book: Foodborne pathogenic microorganisms and natural toxins handbook. Choice Rev. Online.

[4] Veras J.F., Carmo L.S.D., Tong L.C., Shupp J.W., Cummings C., Dos Santos D.A., Cerqueira M.M.O.P., Cantini A., Nicoli J., Jett M. (2008). A study of the enterotoxigenicity of coagulase-negative and coagulase-positive staphylococcal isolates from food poisoning outbreaks in Minas Gerais, Brazil. Int. J. Infect. Dis..

[5] Piette A., Verschraegen G. (2009). Role of coagulase-negative staphylococci in human disease. Veter Microbiol..

[6] Mazzariol A., Cascio G.L., Kocsis E., Maccacaro L., Fontana R., Cornaglia G. (2011). Outbreak of linezolid-resistant Staphylococcus haemolyticus in an Italian intensive care unit. Eur. J. Clin. Microbiol. Infect. Dis..

[7] Brito D.V.D.D., von Dolinger E.J., Abdallah V.O., Darini A.L.C., Filho P.P.G. (2009). Two outbreaks of mixed etiology associated with central venous catheters inserted by phlebotomy in critical neonates. Braz. J. Infect. Dis..

[8] Gentilini E., Denamiel G., Betancor A., Rebuelto M., Fermepin M.R., de Torres R. (2002). Antimicrobial Susceptibility of Coagulase-Negative Staphylococci Isolated from Bovine Mastitis in Argentina. J. Dairy Sci..

[9] Donlan R.M. (2001). Biofilm Formation: A Clinically Relevant Microbiological Process. Clin. Infect. Dis..

[10] Araújo P., Lemos M., Mergulháo F., Melo L., Simões M., Méndez-Vilas A. (2011). Antimicrobial resistance to disinfectants in biofilms. Science Against Microbial Pathogens: Communicating Current Research and Technological Advances.

[11] Foka A., Chini V., Petinaki E., Kolonitsiou F., Anastassiou E.D., Dimitracopoulos G., Spiliopoulou I. (2006). Clonality of slime-producing methicillin-resistant coagulase-negative staphylococci disseminated in the neonatal intensive care unit of a university hospital. Clin. Microbiol. Infect..

[12] Pereira V., Lopes C., Castro A.I., Silva J., Gibbs P., Teixeira P. (2009). Characterization for enterotoxin production, virulence factors, and antibiotic susceptibility of Staphylococcus aureus isolates from various foods in Portugal. Food Microbiol..

[13] Fooladi A.I., Tavakoli H., Naderi A. (2010). Detection of enterotoxigenic Staphylococcus aureus isolates in domestic dairy products. Iran. J. Microbiol..

[14] Shan B., Cai Y.-Z., Brooks J.D., Corke H. (2007). The in vitro antibacterial activity of dietary spice and medicinal herb extracts. Int. J. Food Microbiol..

[15] Dorman H.J.D., Deans S.G. (2000). Antimicrobial agents from plants: Antibacterial activity of plant volatile oils. J. Appl. Microbiol..

[16] Landers T., Cohen B., Wittum T.E., Larson E.L. (2012). A Review of Antibiotic Use in Food Animals: Perspective, Policy, and Potential. Public Heal. Rep..

[17] Lee N.-K., Paik H.-D. (2016). Status, Antimicrobial Mechanism, and Regulation of Natural Preservatives in Livestock Food Systems. Food Sci. Anim. Resour..

[18] Al-Juhaimi F.Y., Ghafoor K., Özcan M.M., Jahurul M.H.A., Babiker E.E., Jinap S., Sahena F., Sharifudin M.S., Zaidul I.S.M. (2018). Effect of various food processing and handling methods on preservation of natural antioxidants in fruits and vegetables. J. Food Sci. Technol..

[19] Pisoschi A.M., Pop A., Georgescu C., Turcuş V., Olah N.K., Mathe E. (2018). An overview of natural antimicrobials role in food. Eur. J. Med. Chem..

[20] Fernebro J. (2011). Fighting bacterial infections—Future treatment options. Drug Resist. Updat..

[21] Kutateladze M., Adamia R. (2010). Bacteriophages as potential new therapeutics to replace or supplement antibiotics. Trends Biotechnol..

[22] Moye Z.D., Woolston J., Sulakvelidze A. (2018). Bacteriophage Applications for Food Production and Processing. Viruses.

[23] Sulakvelidze A. (2013). Using lytic bacteriophages to eliminate or significantly reduce contamination of food by foodborne bacterial pathogens. J. Sci. Food Agric..

[24] Grant A., Parveen S., Schwarz J., Hashem F., Vimini B. (2017). Reduction of Salmonella in ground chicken using a bacteriophage. Poult. Sci..

[25] Soffer N., Woolston J., Li M., Das C., Sulakvelidze A. (2017). Bacteriophage preparation lytic for Shigella significantly reduces Shigella sonnei contamination in various foods. PLoS ONE.

[26] Zampara A., Sørensen M.C.H., Elsser-Gravesen A., Brøndsted L. (2017). Significance of phage-host interactions for biocontrol of Campylobacter jejuni in food. Food Control..

[27] Magin V., Garrec N., Andrés Y. (2019). Selection of Bacteriophages to Control In Vitro 24 h Old Biofilm of Pseudomonas Aeruginosa Isolated from Drinking and Thermal Water. Viruses.

[28] Islam S., Zhou Y., Liang L., Nime I., Liu K., Yan T., Wang X., Li J. (2019). Application of a Phage Cocktail for Control of Salmonella in Foods and Reducing Biofilms. Viruses.

[29] Harper D.R., Parracho H.M.R.T., Walker J., Sharp R., Hughes G., Werthén M., Lehman S., Morales S. (2014). Bacteriophages and Biofilms. Antibiotics.

[30] Abedon S. (2015). Ecology of Anti-Biofilm Agents II: Bacteriophage Exploitation and Biocontrol of Biofilm Bacteria. Pharmaceutical.

[31] Cha Y., Chun J., Son B., Ryu S. (2019). Characterization and Genome Analysis of Staphylococcus aureus Podovirus CSA13 and Its Anti-Biofilm Capacity. Viruses.

[32] Jończyk E., Kłak M., Międzybrodzki R., Górski A. (2011). The influence of external factors on bacteriophages. Rev. Folia Microbiol..

[33] Olson M.R., Axler R.P., Hicks R.E. (2004). Effects of freezing and storage temperature on MS2 viability. J. Virol. Methods.

[34] Brovko L., Anany H., Griffiths M.W. (2012). Bacteriophages for Detection and Control of Bacterial Pathogens in Food and Food-Processing Environment. Adv. Food Nutr. Res..

[35] Greer G.G. (2005). Bacteriophage Control of Foodborne Bacteria. J. Food Prot..

[36] O’Flaherty S., Ross R.P., Meaney W., Fitzgerald G.F., Elbreki M.F., Coffey A. (2005). Potential of the Polyvalent Anti-Staphylococcus Bacteriophage K for Control of Antibiotic-Resistant Staphylococci from Hospitals. Appl. Environ. Microbiol..

[37] Yu P., Mathieu J., Li M., Dai Z., Alvarez P.J.J. (2015). Isolation of Polyvalent Bacteriophages by Sequential Multiple-Host Approaches. Appl. Environ. Microbiol..

[38] Filho R.L.A., Higgins J.P., Higgins S.E., Gaona G., Wolfenden A.D., Tellez G., Hargis B. (2007). Ability of Bacteriophages Isolated from Different Sources to Reduce Salmonella enterica Serovar Enteritidis In Vitro and In Vivo. Poult. Sci..

[39] Bielke L., Higgins S., Donoghue A., Hargis B., Donoghue D. (2007). Salmonella Host Range of Bacteriophages That Infect Multiple Genera. Poult. Sci..

[40] Pamuk Ş., Eker E., Yıldırım Y. (2010). Antibiotic resistance of coagulase negative staphylococci isolated from buffalo milk and some milk products. Kocatepe Vet. J..

[41] Nunes R.S.C., Del Aguila E.M., Paschoalin V.M.F. (2015). Safety Evaluation of the Coagulase-Negative Staphylococci Microbiota of Salami: Superantigenic Toxin Production and Antimicrobial Resistance. BioMed Res. Int..

[42] Sieuwerts S., de Bok F., Mols E., de Vos W., Vlieg J.V.H. (2008). A simple and fast method for determining colony forming units. Lett. Appl. Microbiol..

[43] Holt J.G., Krieg N.R., Sneath P.H.A., Staley J.T., Williams S.T. (2000). Bergey’s Manuel of Determinative Bacteriology.

[44] Zhang M., Li J., Lin H., Zhang W., Lin M., Wu L., Liu W., Mu J., Ye J., Cui X. (2014). Diagnostic value of cytological and microbiological methods in cryptococcal meningitis. Genet. Mol. Res..

[45] Cheesbrough M. (2006). District Laboratory Practice in Tropical Countries by Monica Cheesbrough.

[46] Bauer A.W., Kirby W.M.M., Sherris J.C., Turck M. (1966). Antibiotic Susceptibility Testing by a Standardized Single Disk Method. Am. J. Clin. Pathol..

[47] National Committee for Clinical Laboratory Standards (NCCLS/CLSI) (2007). Performance Standards for Antimicrobial Susceptibility Testing.

[48] Bannerman T.L., Kleeman K.T., Kloos W.E. (1993). Evaluation of the Vitek Systems Gram-Positive Identification card for species identification of coagulase-negative staphylococci. J. Clin. Microbiol..

[49] Funke G., Funke-Kissling P. (2005). Performance of the New VITEK 2 GP Card for Identification of Medically Relevant Gram-Positive Cocci in a Routine Clinical Laboratory. J. Clin. Microbiol..

[50] Rokbani K.B., Ben Abdallah F., Ellafi A., Bakhrouf A. (2011). Adherence assays and slime production of Staphylococcus aureus strains after their incubation in seawater microcosms. Ann. Microbiol..

[51] Stepanović S., Vuković D., Holá V., di Bonaventura G., Djukic S., Cirkovic I., Ruzicka F. (2007). Quantification of biofilm in microtiter plates: overview of testing conditions and practical recommendations for assessment of biofilm production by staphylococci. APMIS.

[52] Borrego J.J., Moriñigo M.Á., de Vicente A., Córnax R., Romero P. (1987). Coliphages as an indicator of faecal pollution in water. Its relationship with indicator and pathogenic microorganisms. Water Res..

[53] Capra M., Quiberoni A., Reinheimer J. (2004). Thermal and chemical resistance of Lactobacillus casei and Lactobacillus paracasei bacteriophages. Lett. Appl. Microbiol..

[54] Kaur S., Harjai K., Chhibber S. (2012). Methicillin-Resistant Staphylococcus aureus Phage Plaque Size Enhancement Using Sublethal Concentrations of Antibiotics. Appl. Environ. Microbiol..

[55] Sangha K.K., Kumar B.V.S., Agrawal R.K., Deka D., Verma R. (2014). Proteomic Characterization of Lytic Bacteriophages of Staphylococcus aureus Isolated from Sewage Affluent of India. Int. Sch. Res. Not..

[56] Mirzaei M.K., Nilsson A.S. (2015). Correction: Isolation of Phages for Phage Therapy: A Comparison of Spot Tests and Efficiency of Plating Analyses for Determination of Host Range and Efficacy. PLoS ONE.

[57] Huang C., Shi J., Ma W., Li Z., Wang J., Li J., Wang X. (2018). Isolation, characterization, and application of a novel specific Salmonella bacteriophage in different food matrices. Food Res. Int..

[58] Wang Y., Wang W., Lv Y., Zheng W., Mi Z., Pei G., An X., Xu X., Han C., Liu J. (2014). Characterization and complete genome sequence analysis of novel bacteriophage IME-EFm1 infecting Enterococcus faecium. J. Gen. Virol..

[59] Ackermann H.-W. (2012). Bacteriophage Electron Microscopy.

[60] Accolas J.-P., Spillmann H. (1979). The Morphology of Six Bacteriophages of Streptococcus thermophilus. J. Appl. Bacteriol..

[61] Philipson L., Albertsson P., Frick G. (1960). The purification and concentration of viruses by aqueous polymer phase systems. Virology.

[62] Jamalludeen N., Johnson R.P., Friendship R., Kropinski A.M., Lingohr E.J., Gyles C.L. (2007). Isolation and characterization of nine bacteriophages that lyse O149 enterotoxigenic Escherichia coli. Veter Microbiol..

[63] Else T.A., Pantle C.R., Amy P.S. (2003). Boundaries for Biofilm Formation: Humidity and Temperature. Appl. Environ. Microbiol..

[64] Kostaki M., Chorianopoulos N., Braxou E., Nychas G.-J.E., Giaouris E. (2012). Differential Biofilm Formation and Chemical Disinfection Resistance of Sessile Cells of Listeria monocytogenes Strains under Monospecies and Dual-Species (with Salmonella enterica) Conditions. Appl. Environ. Microbiol..

[65] O’Flynn G., Ross R.P., Fitzgerald G.F., Coffey A. (2004). Evaluation of a Cocktail of Three Bacteriophages for Biocontrol of Escherichia coli O157:H7. Appl. Environ. Microbiol..

[66] Anany H., Lingohr E.J., Villegas A., Ackermann H.W., She Y.-M., Griffiths M.W., Kropinski A.M. (2011). A Shigella boydii bacteriophage which resembles Salmonella phage ViI. Virol. J..

[67] Food and Drug Administration (FDA) (2002). Bacteriological Analytical Manual.

[68] Spricigo D.A., Bardina C., Cortés P., Llagostera M. (2013). Use of a bacteriophage cocktail to control Salmonella in food and the food industry. Int. J. Food Microbiol..

[69] Tomat D., Casabonne C., Aquili V., Balagué C., Quiberoni A. (2018). Evaluation of a novel cocktail of six lytic bacteriophages against Shiga toxin-producing Escherichia coli in broth, milk and meat. Food Microbiol..

[70] Bennett R.W., Lancette G.A. (2001). Chapter 12: Staphylococcus aureus. Bacteriological Analytical Manual Online.

[71] Sofy A.R., Aboseidah A.A., El-Morsi E.-S., Azmy H.A., Hmed A.A. (2020). Evaluation of antibacterial and antibiofilm activity of new antimicrobials as an urgent need to counteract stubborn multidrug-resistant bacteria. J. Pure Appl. Microbiol..

[72] Gillespie B., Headrick S., Boonyayatra S., Oliver S. (2009). Prevalence and persistence of coagulase-negative Staphylococcus species in three dairy research herds. Veter Microbiol..

[73] Elshaarawy R.F.M., Mustafa F.H.A., Sofy A.R., Hmed A.A., Janiak C. (2019). A new synthetic antifouling coatings integrated novel aminothiazole-functionalized ionic liquids motifs with enhanced antibacterial performance. J. Environ. Chem. Eng..

[74] Jett M., Ionin B., Das R., Neill R., Sussman M. (2001). The staphylococcal enterotoxins. Molecular Medical Microbiology.

[75] Kırkan Ş., Göksoy E.Ö., Kaya O. (2005). Identification and antimicrobial susceptibility of Staphylococcus aureus and coagulase negative staphylococci from bovine mastitis in the Aydın region of Turkey. Turk. J. Vet. Anim. Sci..

[76] Ruaro A., Andrighetto C., Torriani S., Lombardi A. (2013). Biodiversity and characterization of indigenous coagulase-negative staphylococci isolated from raw milk and cheese of North Italy. Food Microbiol..

[77] Guran H.S., Kahya S. (2015). Species Diversity and Pheno-and Genotypic Antibiotic Resistance Patterns of Staphylococci Isolated from Retail Ground Meats. J. Food Sci..

[78] Kenar B., Kuyucuoǵlu Y., Şeker E. (2012). Antibiotic susceptibility of coagulase-negative staphylococci isolated from bovine subclinical mastitis in Turkey. Pak. Vet. J..

[79] Irlinger F. (2008). Safety assessment of dairy microorganisms: Coagulase-negative staphylococci. Int. J. Food Microbiol..

[80] Sawant A., Gillespie B., Oliver S. (2009). Antimicrobial susceptibility of coagulase-negative Staphylococcus species isolated from bovine milk. Veter Microbiol..

[81] Wang C., Li M., Dong D., Wang J., Ren J., Otto M., Gao Q. (2007). Role of Clp in biofilm formation and virulence of Staphylococcus epidermidis. Microbes Infect..

[82] Cucarella C., Solano C., Valle J., Amorena B., Lasa I., Penadés J.R. (2001). Bap, a Staphylococcus aureus Surface Protein Involved in Biofilm Formation. J. Bacteriol..

[83] Tormo-Mas M., Knecht E., Götz F., Lasa I., Penadés J.R. (2005). Bap-dependent biofilm formation by pathogenic species of Staphylococcus: Evidence of horizontal gene transfer?. Microbiology.

[84] Jensen K.C., Hair B.B., Wienclaw T.M., Murdock M.H., Hatch J.B., Trent A.T., White T.D., Haskell K.J., Berges B. (2015). Isolation and Host Range of Bacteriophage with Lytic Activity against Methicillin-Resistant Staphylococcus aureus and Potential Use as a Fomite Decontaminant. PLoS ONE.

[85] Xu J., Chen M., He L., Zhang S., Ding T., Yao H., Lu C. (2015). Isolation and characterization of a T4-like phage with a relatively wide host range within Escherichia coli. J. Basic Microbiol..

[86] Uchiyama J., Rashel M., Maeda Y., Takemura I., Sugihara S., Akechi K., Muraoka A., Wakiguchi H., Matsuzaki S. (2008). Isolation and characterization of a novel Enterococcus faecalis bacteriophage EF24C as a therapeutic candidate. FEMS Microbiol. Lett..

[87] Lin L., Han J., Ji X., Hong W., Huang L., Wei Y. (2011). Isolation and characterization of a new bacteriophage MMP17 from Meiothermus. Extremophiles.

[88] Anand T., Vaid R.K., Bera B.C., Barua S., Riyesh T., Virmani N., Yadav N., Malik P. (2015). Isolation and characterization of a bacteriophage with broad host range, displaying potential in preventing bovine diarrhoea. Virus Genes.

[89] Deghorain M., Bobay L.-M., Smeesters P.R., Bousbata S., Vermeersch M., Perez-Morga D., Drèze P.-A., Rocha E.P.C., Touchon M., Van Melderen L. (2012). Characterization of Novel Phages Isolated in Coagulase-Negative Staphylococci Reveals Evolutionary Relationships with Staphylococcus aureus Phages. J. Bacteriol..

[90] Ly-Chatain M.H. (2014). The factors affecting effectiveness of treatment in phages therapy. Front. Microbiol..

[91] Jault P., Leclerc T., Jennes S., Pirnay J.-P., Que Y.-A., Resch G., Rousseau A.F., Ravat F., Carsin H., Le Floch R. (2019). Efficacy and tolerability of a cocktail of bacteriophages to treat burn wounds infected by Pseudomonas aeruginosa: A randomised, controlled, double-blind phase 1/2 trial. Lancet Infect. Dis..

[92] Pires D.P., Oliveira H., Melo L.D.R., Cerqueira M.A., Azeredo J. (2016). Bacteriophage-encoded depolymerases: their diversity and biotechnological applications. Appl. Microbiol. Biotechnol..

[93] Leskinen K., Tuomala H., Wicklund A., Horsma-Heikkinen J., Kuusela P., Skurnik M., Kiljunen S. (2017). Characterization of vB_SauM-fRuSau02, a Twort-Like Bacteriophage Isolated from a Therapeutic Phage Cocktail. Viruses.

[94] Lu T.K., Koeris M.S. (2011). The next generation of bacteriophage therapy. Curr. Opin. Microbiol..

[95] Chan B.K., Abedon S. (2012). Phage Therapy Pharmacology. Adv. Appl. Microbiol..

[96] Cerca N., Gomes F., Bento J.C., França Â., Rolo J., Miragaia M., Teixeira P., Oliveira R. (2013). Farnesol induces cell detachment from established S. epidermidis biofilms. J. Antibiot..

[97] Hyman P., Abedon S.T. (2010). Bacteriophage Host Range and Bacterial Resistance. Adv. Appl. Microbiol..

[98] Fookes M., Schroeder G., Langridge G.C., Blondel C.J., Mammina C., Connor T.R., Seth-Smith H., Vernikos G.S., Robinson K.S., Sanders M. (2011). Salmonella bongori Provides Insights into the Evolution of the Salmonellae. PLoS Pathog..

[99] Loessner M.J. (2005). Bacteriophage endolysins—current state of research and applications. Curr. Opin. Microbiol..

[100] Rodriguez-Rubio L., Martínez B., Donovan D.M., Rodriguez A., García-Delgado P. (2012). Bacteriophage virion-associated peptidoglycan hydrolases: Potential new enzybiotics. Crit. Rev. Microbiol..

[101] Oliveira H., Melo L.D.R., Santos S.B., Nobrega F.L., Ferreira E.C., Cerca N., Azeredo J., Kluskens L.D. (2013). Molecular Aspects and Comparative Genomics of Bacteriophage Endolysins. J. Virol..

[102] Santos S.B., Carvalho C., Azeredo J., Ferreira E.C. (2014). Population Dynamics of a Salmonella Lytic Phage and Its Host: Implications of the Host Bacterial Growth Rate in Modelling. PLoS ONE.

[103] Lavigne R., Darius P., Summer E.J., Seto D., Mahadevan P., Nilsson A.S., Ackermann H.-W., Kropinski A.M. (2009). Classification of Myoviridae bacteriophages using protein sequence similarity. BMC Microbiol..

[104] Bigwood T., Hudson J., Billington C. (2009). Influence of host and bacteriophage concentrations on the inactivation of food-borne pathogenic bacteria by two phages. FEMS Microbiol. Lett..

[105] El-Shibiny A., El-Sahhar S., Adel M. (2017). Phage applications for improving food safety and infection control in Egypt. J. Appl. Microbiol..

[106] Duc H.M., Son H.M., Honjoh K.-I., Miyamoto T. (2018). Isolation and application of bacteriophages to reduce Salmonella contamination in raw chicken meat. LWT.

[107] Hooton S.P., Atterbury R.J., Connerton I. (2011). Application of a bacteriophage cocktail to reduce Salmonella Typhimurium U288 contamination on pig skin. Int. J. Food Microbiol..

[108] Abedon S. (2011). Phage Therapy Pharmacology. Adv. Appl. Microbiol..

[109] Klumpp J., Lavigne R., Loessner M.J., Ackermann H.-W. (2010). The SPO1-related bacteriophages. Arch. Virol..

[110] Łobocka M., Hejnowicz M.S., Dąbrowski K., Gozdek A., Kosakowski J., Witkowska M., Ulatowska M.I., Weber-Dąbrowska B., Kwiatek M., Parasion S. (2012). Genomics of Staphylococcal Twort-like Phages-Potential Therapeutics of the Post-Antibiotic Era. Adv. Appl. Microbiol..

[111] Fauquet C.M., Mayo M.A., Maniloff J., Desselberger U., Ball L.A. (2005). Virus Taxonomy.

[112] Maura D., Morello E., Du Merle L., Bomme P., Le Bouguénec C., Debarbieux L. (2011). Intestinal colonization by enteroaggregative Escherichia coli supports long-term bacteriophage replication in mice. Environ. Microbiol..

[113] Langlet J., Gaboriaud F., Gantzer C. (2007). Effects of pH on plaque forming unit counts and aggregation of MS2 bacteriophage. J. Appl. Microbiol..

[114] Chan B.K., Abedon S. (2015). Bacteriophages and their enzymes in biofilm control. Curr. Pharm. Des..

[115] Flemming H.-C., Wingender J. (2010). The biofilm matrix. Nat. Rev. Genet..

[116] Briandet R., Lacroix-Gueu P., Renault M., Lecart S., Meylheuc T., Bidnenko E., Steenkeste K., Bellon-Fontaine M.-N., Fontaine-Aupart M.-P. (2008). Fluorescence Correlation Spectroscopy To Study Diffusion and Reaction of Bacteriophages inside Biofilms. Appl. Environ. Microbiol..

[117] Hu J., Miyanaga K., Tanji Y. (2010). Diffusion properties of bacteriophages through agarose gel membrane. Biotechnol. Prog..

[118] Wilking J.N., Angelini T.E., Seminara A., Brenner M.P., Weitz D.A. (2011). Biofilms as complex fluids. MRS Bull..

[119] Pennone V., Gaitero M.S., O’Connor P., Coffey A., Jordan K., Van Raaij M.J., McAuliffe O., Gaitero S., Connor O., Raaij V. (2019). Inhibition of L. Monocytogenes Biofilm Formation by the Amidase Domain of the Phage vB_LmoS_293 Endolysin. Viruses.

[120] Shen Y., Mitchell M.S., Donovan D.M., Nelson D.C. (2012). Phage-based enzybiotics. Bacteriophages in Health and Disease.

[121] Guenther S., Herzig O., Fieseler L., Klumpp J., Loessner M.J. (2012). Biocontrol of Salmonella Typhimurium in RTE foods with the virulent bacteriophage FO1-E2. Int. J. Food Microbiol..

[122] Bao H., Zhang P., Zhang H., Zhou Y., Zhang L., Wang R. (2015). Bio-Control of Salmonella Enteritidis in Foods Using Bacteriophages. Viruses.

